# Sexual Knowledge, Desires, and Experience of Adolescents and Young Adults With an Autism Spectrum Disorder: An Exploratory Study

**DOI:** 10.3389/fpsyt.2021.685256

**Published:** 2021-06-09

**Authors:** Christian C. Joyal, Julie Carpentier, Suzie McKinnon, Claude L. Normand, Marie-Hélène Poulin

**Affiliations:** ^1^Department of Psychology, Université du Québec à Trois-Rivières, Trois-Rivières, QC, Canada; ^2^Department of Psychoeducation, Université du Québec à Trois-Rivières, Trois-Rivières, QC, Canada; ^3^Integrated University Health and Social Services Centre (IUHSSC) of Saguenay-Lac-Saint-Jean, IUHSSC Bas-Saint-Laurent and IUHSSC Côte-Nord, Saguenay, QC, Canada; ^4^Intellectual Disability and Autism Spectrum Disorder Research Institute, IUHSSC of Mauricie and Centre-du-Québec, Trois-Rivières, QC, Canada; ^5^Department of Psychoeducation and Psychology, Université du Québec en Outaouais, Gatineau, QC, Canada; ^6^Department of Psychoeducation, Université du Québec en Abitibi-Témiscamingue, Rouyn-Noranda, QC, Canada

**Keywords:** autism spectrum disorder, sexuality, adolescent, asperger, young adult

## Abstract

Although most persons with an Autism Spectrum Disorder (ASD) wish to have romantic and/or sexual relationships, little is known about self-report sexuality of adolescents/young adults with ASD. In this exploratory study, 172 male and female adolescents/young adults (68 with ASD and 104 without ASD) completed an online version of the Sexual Behavior Scale-Third edition. Although many more similarities than differences were observed between the groups for views and desires about romantic relationships (e.g., wishing to have a girlfriend/boyfriend), fewer participants with ASD (mostly boys) had experience with a variety of sexual/dyadic behaviors, and approximately half of girls with ASD reported negative sexual experiences. Significantly higher rates of participants with ASD felt their knowledge about sexuality was limited and found it difficult to understand sexual education compared with typically developing (TD) participants. Significantly lower rates of participants with ASD reported that they identify to their assigned gender compared with TD participants. Multiple regressions revealed that being older at first diagnosis and possessing better knowledge about sexuality were significant predictors of both positive and negative sexual experience. This study explores strengths and challenges related with the sexual health of adolescents/young adults with ASD and implications for clinical and educational practice are discussed.

## Introduction

Adults with Autism Spectrum Disorder (ASD) have particular challenges to face in their attempts to be involved in romantic/sexual relationships and they engage, on average, in fewer socio-sexual behaviors than typically developing (TD) individuals (1–3). Personal (e.g., difficulties in social cognition), institutional (e.g., insufficient sexual education), and societal (e.g., ableism, assumptions, stigmatization, and exclusion) barriers contribute to limit the sexual knowledge of people with ASD (especially adolescents) and lower their chances to have romantic/sexual relationships (2). This type of data is of utmost importance as it helps developing education or training plans based on specific needs of persons with ASD to initiate and maintain romantic/sexual relationships. However, available studies are mostly limited to adults and information gathered from adolescents/young adults with ASD is warranted.

Adolescence and the transition into adulthood represent a challenging developmental phase involving important bio-psycho-social modifications (including physical, emotional, social, and cognitive transformations) and intense learning/adaptation (4). One major challenge faced by most adolescents, with or without ASD, is the discovery and exploration of personal and interpersonal sexuality (5). Adolescent sexuality is particularly complex because it is a multidimensional process involving areas as diverse as expanding sexual knowledge (about oneself and others), exploring sexual preference, wanting (or not) to develop intimate partnerships, fulfilling (or not) important socio-sexual needs (e.g., being liked and accepted, giving and receiving affection, feeling attractive, sharing feelings) and confirming (or questioning) gender identity and sexual orientation (6). Although transition into adulthood in general and sexuality in particular are challenging for most adolescents, it might be even more so for those with ASD (7).

Contrary to traditional belief [e.g., (8)], the majority of persons with ASD clearly express desire to have romantic/sexual relationships [e.g., (9), see (1, 10) for meta-analyses]. However, available studies about sexual health of persons with ASD are typically based on adult participants or indirect reports from parents or counselors of adolescents, often without comparative data. Given the importance of romantic/sexual exploration and sexual health during adolescence in general and the paucity of data obtained directly from young persons with ASD, the main goal of this exploratory study was to directly ask adolescents and emerging adults with ASD about their sexual knowledge, desires, challenges and experience.

### Sexual Knowledge, Desires, and Experience of Adolescents/Young Adults With ASD

In opposition to the classic assumption that they are too naïve and immature to be interested in sexuality [e.g., (11)], a growing number of studies suggest that most adolescents/young adults with ASD (but not all), just like any person their age, have socio-sexual interests, including for dyadic relationships and behaviors (12–16). According to their parents, adolescents/young adults with ASD have, on average, less sexual and privacy knowledge, and they engage less in socially appropriate (and more in inappropriate) sexual behaviors than typically developing (TD) adolescents [e.g., (13, 15)]. These conclusions are uncertain, however, because parents of children with ASD tend to overestimate their own knowledge about their child's sexual life (17). In addition, these parents tend to underestimate the sexual experience and activities of their child (18–20). As stressed by Dekker et al. (18), underestimation of sexual behaviors by these parents may reflect not only the fact that most of these behaviors are performed privately, but also that parents might assume that sexuality is non-existent or irrelevant for their child with ASD. Therefore, self-report measures are preferable for the study of sexual desires and behaviors of persons with ASD (21), including adolescents/young adults.

Few studies limited to adolescents or younger adults (i.e., 15–25 y.o.) with ASD have used direct assessment (self-report) of their sexual/romantic knowledge, desires and experience (18, 22, 23). The majority of these participants express interests in sexuality and want to be involved in a romantic relationship. When a comparison group of TD peers is included, more similitudes than differences are found for sexual knowledge and behaviors compared with adolescents/young adults with ASD. As reported below, these studies contain limitations addressed by the present investigation. Dewinter et al. (23) surveyed male adolescents with (*n* = 50 without intellectual deficits, mean age of 16.7 years, 15–18 y.o.) or without (*n* = 90 matched on age, educational level, and ethnicity) ASD and found very similar rates of sexual experience between the groups. These interesting results, at odds with most hypotheses based on parent and caregiver reports, might reflect reality, being based on self-reports. However, they might also apply only to younger boys. Approximately 2 years later, a subset of the same participants with ASD (*n* = 30, mean age of 18.6 years) had significantly less sexual experience than TD boys in dyadic behaviors [i.e., partnered sexual behaviors, French kissing, and petting; (24)]. Therefore, adolescents with ASD may be just as sexually active, but have less dyadic sexual experience than their peers.

In another comparative study of self-reported (and parent reported) sexuality of adolescents/young adults with (*n* = 58, mean age of 16.8 years, 13–21 y.o.) and without (*n* = 91, mean age of 16.3 years) ASD, Dekker et al. (18) found, again, no significant differences between the groups for sexual desires and sexual behaviors. However, the Bonferroni approach (α/number of comparisons) was adopted to correct multiple comparisons in that study, shrinking the level of significance to a very conservative level (0.0008), thus enhancing the risk of committing type-II errors (25). As acknowledged by the authors, “adolescents with ASD have shown approximately one-third of the sexual behaviors and the TD adolescents about half of the sexual behaviors [of the 41 item sexual/intimate subscale]” (p. 1,732). Clearly, the two groups seem to differ in that study, which deserves further self-report investigation.

Taken together, these two comparative studies based on self-reports concluded much more similarity than difference between groups for sexual experience (18, 23). However, the fact that the mean age of participants was relatively low in both studies (16.3 years for TD participants in each study) might have induced a ceiling effect for sexual experience. The absence of inter-group differences might also reflect the fact that both studies were conducted in the Netherlands where, perhaps, sexual education provided to adolescents with ASD is better than in other countries. Clearly, there is a need to investigate sexual knowledge and experience with older adolescents/young adults with ASD outside the Netherlands.

A third investigation on sexual health of adolescents with ASD based on self-report was a qualitative study with 27 participants conducted in the U.S.A. (22). Female participants were more likely than males to report no interest in sexuality and to express fear of being taken advantage of. There were only seven girls in that study, however.

Finally, Fernandes et al. (12) and Hartmann et al. (20) compared responses of adolescents/young adults with ASD to those of their parents. Both studies confirmed that most participants report having sexual interest. Fernandes et al. also found a relatively high rate of participant with paraphilic interests (24%), associated with symptom severity (higher) and intellectual capacity (lower). Hartmann et al. assessed an online survey to 100 young adults (18–30 y.o.) with ASD. They confirmed that the rate of romantic interest was relatively high (73%) and knowledge about basic sexual education (e.g., knowing about sexual transmitted diseases) and privacy (e.g., aware of social rules about undressing in private) was good. Still, almost half (46%) of these participants with ASD reported that they never or rarely seek privacy when they masturbate, with approximately a third (36%) reporting the same for using the toilet. As stressed by the authors, these results suggest the need for better sexual education among adolescents with ASD. In addition, nearly two-thirds of that sample reported at least one item of both the sexual victimization (62%) and sexual aggression (65%) scales. However, this study did not compare rates between genders.

Overall, four of these self-report investigations about sexuality of adolescents/young adults with ASD mostly concerned boys (12, 18, 22, 23) and the fifth did not distinguish between genders (20). More data, especially self-reported, concerning sexual knowledge, desires, and experiences of female adolescents/young adults with ASD are needed, along with comparative data from TD adolescents.

### ASD and Sexual Victimization

Individuals with ASD seem to be at higher risk for sexual victimization than individuals without ASD, although evidence-based data are still scarce on that matter [see (26, 27) for reviews]. In adults with ASD, a handful of studies are available, indicating high prevalence of sexual victimization, ranging between 46 and 78% (20, 28–32). Not all studies included a comparison group, however. On one study, for instance, the proportion of female college students women reporting unwanted sexual contact on campus was in fact lower among those with ASD compared with those without ASD, although rates were alarmingly high in both groups [61.5 and 83.2%, respectively; (28)]. In young adults with ASD (18–30 years), two independent studies reported a lifetime prevalence of 62% for sexual victimization (20, 31). Earlier data indicated that odds ratios for sexual victimization were equally elevated for men and women with ASD (29), although more recent studies suggested that women with ASD are at significantly higher risks than men with ASD (30). Accordingly, parents of adolescent girls with ASD commonly worry about the risks of sexual exploitation because they feel their child is overly trusting of others (33). Homosexuality could further elevate these risks (34), just as it is the case in the general population [e.g., (35)].

Data concerning the sexual victimization of adolescents/young adults with ASD are also scarce. A prospective Swedish investigation reported that at 18 years old girls but not boys with ASD were significantly more likely to have been sexually victimized than minors without ASD (36). In a Canadian study, rates of sexual victimization before adulthood were similar (55.6 vs. 50%, respectively) between persons with and without ASD (32). Still, odds for sexual assault by a peer were 7.3 higher for those with ASD compared with those without ASD (32). In the same study, rates of sexual victimization during adulthood were also similar between the groups (46.7 vs. 40.5%, respectively), although persons with ASD were 3 times more likely to be sexually assaulted by an unknown adult or to be the victim of a rape compared with persons without ASD (32). The relatively low sample sizes in that study prevented comparisons between genders, however. Another study reported a rate of 40.1% for childhood sexual abuse among women with ASD traits, although no comparison group was included (37). Overall, the few existing studies on sexual victimization of persons with ASD suggest that they are at higher risks than the general population. Possible difference between genders for these risks deserves further investigation.

A potentially important factor for sexual victimization of persons with ASD is socio-sexual knowledge. As could be expected, better capacities in the social and communication domains are associated with higher dyadic sexual well-being and higher sexual satisfaction, arousability, and desire in adults with ASD (38). The link between sexual knowledge and sexual well-being among persons with ASD is unclear, however. On one hand, Brown-Lavoie et al. (29) reported that sexual knowledge is significantly and inversely associated with sexual victimization. As stressed by the authors, social skills deficits and lack of sexual knowledge may render individuals with ASD more vulnerable to sexual victimization. It is worth noting, however, that the definition of sexual knowledge in that study was limited to physiological and biological information (i.e., related to sexually transmitted infections, contraception, and reproductive health). On the other hand, the results of a meta-analysis (although based on only nine studies) suggested that females with ASD have both better sexual understanding and more negative sexual experiences than males with ASD (10). It remains possible that relatively better socio-sexual knowledge (e.g., understanding that two lovers can kiss and touch each other under certain circumstances) in persons with ASD increases their risks of being sexually victimized. Given that social and communication abilities are higher, on average, in women than in men with ASD [e.g., (39)], these factors might explain in part gender differences found in risks of sexual victimization.

Another possible risk factor for sexual victimization is age at first diagnosis of ASD. Qualitative data suggest that women with ASD who were diagnosed late (i.e., 15 y.o. or older) are at higher odds to be sexually victimized because they are more likely to be in a relationship or to be interested in dyadic sexuality (40). Therefore, gender, sexual knowledge, and age at first diagnosis might represent significant factors for risks of sexual victimization in adults with ASD.

### ASD and Sexual Education

Formal and informal sexual education is of utmost importance for the sexual health of any adolescent/young adult, let alone those with ASD (41). Although most programs of sexual education for persons with ASD focus more on biological content (e.g., anatomy, puberty, reproduction) and self-awareness/safety (e.g., boundaries, assertiveness, privacy) than personal sexuality (e.g., sexual orientation, masturbation) and relationships (e.g., dating, emotions, dyadic behaviors), they significantly improve outcomes (42). For different reasons [including prejudice and stigmatization; (43)], individuals with ASD usually receive less sexual guidance, support and education than their TD peers (1, 3, 13, 16, 44). Furthermore, when sexual education classes provide specific information about sexuality, students with ASD report that they do not know how to apply it to life situations (44). In addition, persons with ASD learn significantly less about sexuality and romantic functioning from peers and friends (and more from parents) than TD persons, a type of learning more closely related with real life circumstances (16, 29, 44, 45). As a result, sexual knowledge is more limited for persons with ASD, especially during the crucial period of adolescence or the first years of adulthood (1, 18, 20). Accordingly, all adolescents/young adults with ASD who took part in a recent qualitative study (*n* = 27) expressed a need to receive more sexual education (22).

### ASD and Sexual Diversity

Sexual orientation (e.g., non-heterosexuality) and sexual identity (non-cisgender) are more varied or less rigid, on average, in persons with ASD compared with TD individuals [see (26, 27) for reviews]. Rates of same-sex behaviors, homosexual orientation or non-exclusive sexual interests are consistently higher in adults with ASD compared with the general population (14, 34, 38, 46–55). In adolescents with ASD, results are mixed. Although lower rates of exclusive heterosexuality (~60%) are reported by parents or caregivers (12, 14), there is no difference with TD peers according to self-reports (18, 23). Still, adolescents with ASD seem to be more inclined to same-sex sexual interactions than their TD peers (23). These mixed results might reflect different proportions of girls included across studies as the effect is more pronounced among women than among men with ASD (46, 48, 49, 51). Different levels of functioning might also be associated with different level of sexual orientation fluidity [with lower levels of functioning being associated with higher odds of non-heterosexual orientation; (52)].

Significantly higher rates of opposite gender identification, non-binary gender, and gender dysphoria are also found in persons with ASD compared with TD individuals (48, 56–60). Autistic traits are higher in transgender persons (61–64), and they are associated with gender variance in TD children (49, 50, 65).

Self-reports of adults with ASD also indicate higher rates of asexuality (51, 66) and lower levels of sexual drive (46) or sexual desire (67), on average, compared with the general population. Women with ASD report less sexual interest, on average, than men with ASD (30). It remains to be seen whether similar conclusions apply to adolescents/young adults with ASD. Anecdotal evidence based on chart reviews suggest that a significant minority of adolescents with ASD seem to be asexual (68).

### Objectives

The main goal of this exploratory study was to gain information about sexual knowledge, desires, challenges, and experience of adolescents and emerging adults with ASD. An additional objective of this study was to compare responses of participants with ASD to those of a comparison group, as well as responses from boys vs. girls to document the nature and importance of difference (if any) between these groups. Negative sexual experience were also considered to explore whether adolescents/young adults with ASD are at higher risk to be sexually victimized compared with their peers and, if it is the case, which type of sexual victimization is most likely to occur. Finally, this study aims at assessing the value of selected variables (age, gender, age at first diagnosis of ASD, sexual knowledge, level of socialization outside family, and desire to have sex with others) to predict general sexual experience and sexual victimization of adolescents/young adults with ASD.

These data will allow obtaining a general picture of the sexual health of young persons with ASD. These self-reported data will also help identifying areas of sexual satisfaction and dissatisfaction expressed by young persons with ASD, which should enable refining educational and clinical programs accordingly.

### Research Questions

This exploratory study attempted to answer the following research questions: (1) What are the strengths and need of adolescents/young adults with ASD in regard with their sexuality? (2) What are the differences, if any, between adolescents/young adults with and without ASD in regard with their sexuality? (3) What are the differences, if any, between boy and girls with ASD in regard with their sexuality? (4) Which recommendations can be made for professionals working in clinical or educational setting in regard with these results?

### Hypotheses

Given its exploratory nature, this study was based only on three broad hypotheses: (1) Whereas adolescents/young adults with ASD will show interest and desires to have dyadic romantic/sexual relationships, they will report having less knowledge and less experience with these relationships than TD adolescents/young adults; (2) Significantly more girls with ASD than both boys with ASD and TD girls will report having negative sexual experience; (3) Gender (girls), age (higher), and sexual knowledge (higher) will represent significant predictors of dyadic positive sexual experience among participants with ASD.

## Materials and Methods

### Participants

A total of 194 adolescents/young adults began the survey, of which 172 filled all questions included in this study (mean age: 19.2 ± 2.7): 68 with ASD (41 boys, mean age: 19.4 ± 2.9 and 27 girls, mean age: 19.7 ± 2.7) and 104 without ASD (29 boys, mean age: 18.4 ± 2.1 and 75 girls, mean age: 19.1 ± 2.6; no significant difference in mean age between subgroups). No incentive was offered to participate in the study. Academic achievement varied, ranging from primary school (3%), currently attending high school (39%), having a high school diploma (35%), and attending or having a college degree (23%). All participants were French speaking Caucasians recruited in the province of Quebec (Canada). Using G^*^Power (www.gpower.hhu.de), it was determined that 88 participants were necessary to achieve a statistical power of 0.80 based on an effect size of 0.30 and an alpha set at 0.05. Given the high number of bivariate analyses included in this study (see below), that sample size target was doubled. All participants with ASD were recruited in clinical settings, community organizations and academic programs assisting adolescents with autism. They all received formal diagnoses of ASD without intellectual deficits by specialized mental health professionals based on the Diagnostic and Statistical Manual of Mental Disorders [4th ed., Text Revised, DSM; (69)], which included Autistic Disorder and Asperger's Syndrome. Diagnosis confirmation was obtained by self-report from participants.

### Material

An online survey using REDCap was designed based on the Sexual Behavior Scale-Third edition (SBS-III) developed by Stokes and colleagues to evaluate sexual health of persons with ASD (2, 30). The complete paper version of the questionnaire is available elsewhere (70). Following the approach of Pecora et al. (30) and Hancock et al. (2), items of the questionnaire were used individually, not in grouped domains. Given the main purpose of this study, only items related to sociodemographic information, sexuality, romantic relationships, and sexual education were used (see Tables 1–3 for all items retained). Because questions about inadequate or non-optimal social and sexual behaviors have been found to be non-valid in self-reports of adolescent/young adults with ASD [they are underestimated compared with parent reports or not well-understood by participants; (18, 71)], questions about inappropriate behaviors were not included in this study. It took ~20 min on average to complete the survey.

**Table 1:** Prevalence and inter-group comparisons of adolescents/young adults with (*n* = 68) and without (*n* = 104) a diagnosis of ASD^1^.

**Items**	**With ASD agree**	**Without ASD agree**	***p***	***V***
	***n* (%)**	***n* (%)**		
**Socializing**				
I rarely or sometimes socialize with people other than my family	31 (45.6%)	9 (8.7%)	0.001^a^	0.43^**^
**Views about romantic relationships**				
I believe that being in a long-term relationship in the future is important	55 (80.9%)	95 (91.3%)	0.045	0.15
The things that make up a good romantic relationship include if my boyfriend/girlfriend and I:				
Get along	63 (92.7%)	97 (93.3%)	0.859	0.02
Regularly speak with one another	46 (67.6%)	86 (82.7%)	0.022	0.17
Spend a lot of time together	42 (61.8%)	44 (44.3%)	0.013	0.19
Feel emotionally close with each other	46 (67.6%)	80 (76.9%)	0.179	0.10
Kiss, hug, hold hands etc.	36 (52.9%)	70 (67.3%)	0.058	0.14
Engage in sexual behaviors together	24 (35.3%)	57 (54.8%)	0.012	0.19
Trust each other	57 (83.8%)	99 (95.2%)	0.012	0.19
Are affectionate toward each other	55 (80.9%)	92 (88.5%)	0.168	0.11
Speak about our feelings and emotions to each other	46 (67.6%)	91 (87.5%)	0.002	0.24
Go on dates together	40 (58.8%)	67 (64.4%)	0.459	0.06
Have exactly the same interests	44 (64.7%)	39 (37.5%)	0.001	0.27^*^
Do nice things for each other so that it's fair	50 (73.5%)	78 (75.0%)	0.829	0.02
Looks after me and provides for me	50 (73.5%)	87 (83.7%)	0.107	0.12
I look after and provide for my boyfriend/girlfriend	50 (73.5%)	86 (82.7%)	0.149	0.11
Have open and honest conversations with each other	60 (88.2%)	98 (94.2%)	0.160	0.11
**Sexual interests and desires**				
I would like to have a boyfriend or girlfriend in the near future	53 (77.9%)	92 (88.5%)	0.064	0.14
I am, or have been sexually attracted toward someone	46 (67.6%)	100 (96.2%)	0.001	0.39^**^
I would like to have sex with others	46 (67.6%)	98 (94.2%)	0.001	0.35^**^
I want to be in a sexual relationship with another person	41 (60.3%)	89 (85.6%)	0.001	0.29^*^
I think that I will be in a sexual relationship in the future	59 (88.1%)	98 (97.0%)	0.021	0.18
My level of interest in sex and sexual topics is about the same or more than other people my age	41 (60.3%)	95 (91.3%)	0.001	0.37^**^
I never or rarely think about sex and sexual behaviors:	20 (29.4%)	10 (9.6%)	0.001	0.26^*^
**Internet use**				
During the last month:				
I visited a web site devoted to sexual education at least once a week	4 (5.9%)	0 (0.0%)	0.012	0.19
I chatted with someone on a dating website at least once a week	5 (7.4%)	4 (3.8%)	0.313	0.08
I watch pornographic (explicit sexuality) photos or videos at least once a week	21(30.9%)	33 (31.7%)	0.907	0.01
I masturbated watching pornographic (explicit sexuality) photos or videos at least once a week	20 (29.4%)	32 (30.8%)	0.850	0.01
**Sexual strategies and motivations**				
If I wanted to have casual sex (having sex with someone without being in a couple or engaged in a long-term romantic relation), I would:				
Kiss them	21 (30.9%)	36 (34.6%)	0.611	0.04
Talk to them	50 (73.5%)	66 (63.5%)	0.168	0.11
Try to have the same interests as them	23 (33.8%)	10 (9.6%)	0.001	0.29^*^
Give them something nice	22 (32.4%)	10 (9.6%)	0.001	0.29^*^
Sit next to them	30 (44.1%)	36 (34.6%)	0.210	0.10
Watch them almost all of the time	10 (14.7%)	24 (23.1%)	0.178	0.10
Tease them to get their attention	15 (22.1%)	48 (46.2%)	0.001	0.25^*^
Try to be around them almost all of the time	15 (22.1%)	22 (21.2%)	0.888	0.01
Ask them on a date	29 (42.6%)	54 (51.9%)	0.234	0.10
Tell others that I like the person	13 (19.1%)	20 (19.2%)	0.985	0.00
Tell them things about me that I think the other person will like	27 (39.7%)	18 (17.3%)	0.001	0.25^*^
Touch them (arms, back, etc.) to show them I am interested in them	19 (27.9%)	36 (34.6%)	0.359	0.07
The reasons that I might have sex in the future (or have in the past) are:				
Because it feels good (Enjoyment)	33 (48.5%)	83 (79.8%)	0.001	0.33^**^
For fun	36 (52.9%)	81 (77.9%)	0.001	0.26^*^
Because we are friends	2 (2.9%)	8 (7.7%)	0.193	0.10
Because we are in relationships	45 (66.2%)	86 (82.7%)	0.013	0.19
To show that I love them	22 (32.4%)	33 (31.7%)	0.932	0.01
Because I feel like I have to	4 (5.9%)	5 (4.8%)	0.757	0.02
Because we are married	8 (11.8%)	19 (18.3%)	0.252	0.09
Reproduction (to have children)	26 (38.3%)	57 (54.8%)	0.003	0.16
**Sexual/dyadic experience**				
I have held another person's hand (someone that you liked or you were interested in)	47 (69.1%)	93 (89.4%)	0.001	0.26^*^
I have hugged another person	60 (88.2%)	101 (97.1%)	0.020	0.18
I have kissed another person on the lips (mouth closed)	38 (55.9%)	87 (83.7%)	0.001	0.31^**^
I have French kissed someone (includes use of tongue)	26 (38.2%)	81 (77.9%)	0.001	0.40^**^
I have had some form of sexual experiences (e.g., touching another sexually, foreplay, any sexual activity, oral sex, sexual intercourse, etc.)	27 (39.7%)	85 (81.7%)	0.001	0.43^**^
Have you ever had a boyfriend or girlfriend?	39 (58.2%)	82 (78.8%)	0.004	0.22
I enjoy using pornographic materials	32 (47.1%)	58 (55.8%)	0.263	0.09
**Negative sexual experiences/worries**				
I have agreed to have sex with someone and regretted it afterward	18 (26.5%)	35 (33.7%)	0.318	0.08
I have agreed to have sex with someone that I didn't really want to	18 (26.5%)	34 (32.7%)	0.385	0.07
I have been the victim of unwanted sexual advances or behaviors from others	19 (27.9%)	28 (26.9%)	0.884	0.01
I have initiated a sexual behavior with someone, even though I didn't really want to	16 (23.5%)	21 (20.2%)	0.603	0.04
I have been teased or victimized by other people because I didn't know as much about sex as they did	13 (19.1%)	14 (13.5%)	0.319	0.08
I am worried that my sexual behaviors might be misunderstood by other people	26 (38.8%)	17 (16.8%)	0.001	0.25^*^
I worry that I may be taken advantage of	26 (38.8%)	34 (33.7%)	0.496	0.05
**Sexual education and privacy knowledge**				
I don't know much/I would like to know better about sexuality	29 (42.6%)	16 (15.4%)	0.001	0.30^**^
I know about the same/I know much more or much about sexuality than most people my age	39 (57.4%)	88 (84.6%)	0.001	0.34^**^
It was easy for me to understand the sex-education that I received	33 (48.5%)	83 (79.9%)	0.001	0.33^**^
I would like to know more about sexuality and sexual health	39 (57.4%)	63 (60.6%)	0.674	0.03
Who taught you what things and actions should be done in private?				
My parents	42 (61.8%)	53 (51.0%)	0.164	0.11
My brother/s or sister/s	4 (5.9%)	5 (4.8%)	0.757	0.02
My friends	16 (23.5%)	30 (28.8%)	0.441	0.06
My grandparents	4 (5.9%)	5 (4.8%)	0.757	0.02
My teachers	24 (35.3%)	25 (24.0%)	0.110	0.12
My counselor/support worker	19 (27.9%)	8 (7.7%)^*^	0.001	0.27^*^
People on TV or movies	22 (32.4%)	31 (29.8%)	0.724	0.03
I learnt on my own/worked it out for myself	32 (47.1%)	68 (65.4%)	0.017	0.18
I have mostly learnt about sex-related topics from:				
My parents	12 (17.9%)	12 (11.5%)	0.242	0.09
My brother/s or sister/s	2 (3.0%)	0	0.076	0.14
My friends	5 (7.5%)	31 (29.8%)	0.001	0.27^*^
School	26 (38.8%)	25 (24.0%)	0.039	0.16
Reading (books, magazines)	6 (9.0%)	6 (5.8%)	0.426	0.06
Services such as counseling or community program	1 (1.5%)	2 (1.9%)	0.834	0.02
TV/movies/Internet	8 (11.9%)	15 (14.4%)	0.642	0.04
None	4 (6.0%)	8 (7.7%)	0.667	0.03
I have been taught the importance of				
Use contraception (e.g., condoms, contraceptive pill, etc.)	66 (97.1%)	104 (100.0%)	0.079	0.13
Having tests for sexually-transmitted infections (STI's)	60 (88.2%)	96 (92.3%)	0.369	0.07
Not making important decisions about sexual activities while affected by alcohol or drugs	57 (83.8%)	91 (87.5%)	0.496	0.05
The sexual activities that should be engaged in only in privacy are:				
Kissing someone	8 (11.8%)	2 (1.9%)	0.007	0.21
Touching someone in a sexual way	63 (92.6%)	88 (84.6%)	0.116	0.12
Undressing the person	66 (97.1%)	99 (95.2%)	0.545	0.05
Sexual behaviors (other than sexual intercourse)	61 (89.7%)	92 (88.5%)	0.799	0.02
Sexual intercourse	64 (94.1%)	93 (89.4%)	0.286	0.08
**Sexual identity and orientation**				
I clearly identify myself as the gender that I was born (boy or girl)	50 (73.5%)	99 (95.2%)	0.001	0.31^**^
I consider myself as transgender^b^	6 (8.8%)	2 (1.9%)	0.036	0.16
I consider my sexual orientation to be:				
Heterosexual (attracted to people of the opposite sex)	43 (63%)	74 (71.2%)	0.276	0.08
Homosexual (attracted to people of the same sex)	6 (9%)	1 (1.0%)	0.011	0.20
Bisexual (attracted to people of both sex)	10 (14.7%)	20 (19.2)	0.445	0.06
Asexual (attracted to neither sex)	4 (6%)	1 (1.0%)	0.060	0.14
Questioning (not sure who I am attracted to)	5 (7.4%)	8 (7.7%)	0.934	0.01

1 *Autistic Spectrum Disorder*.

*N/A, not applicable*.

a *The lowest possible p-value was set at 0.001*.

b *Reported to be transgender or did not feel that assigned gender was correct*.

* *Near medium effect size (0.25–0.29)*.

** *Medium effect size (0.30 and over)*.

### Statistical Analyses

Following the approach of Hancock et al. (2) and Pecora et al. (30), a series of Chi Squares was first computed to compare responses of participants with and without ASD. Because this approach inflates the risk of Type I error, between-group significance was based solely on effect sizes (Cramer's *V*). The *p*-values were also calculated, but only for information purposes. Given the exploratory nature of this study (and its signaling function), large (>0.50), medium (0.30–0.50) and near medium (0.25–0.29) effect sizes were considered as significant. Statistical power calculation (PASS software; www.ncss.com) revealed that a sample of 172 participants allowed to achieve a statistical power of 0.80 to detect an effect size of 0.26 using 1 degree of freedom Chi-Squares.

In a second step, simple logistic regressions were computed to evaluate the magnitude of variance explained by individual variables for two dichotomized dependent variables (DV: positive and negative sexual experience) related to participants with ASD. Given the number of participants with ASD (*n* = 68), six independent variables per DV were used (for a minimum of 10 participants per independent variable): Gender; age; age at first diagnosis for ASD; level of knowledge about sexuality and sexual behaviors (high level; answering yes to the question: “I know about the same as most people of my age about sexuality” or “I know much more than other people my age about sexuality”), level of socialization outside family (low level; answering yes to “I rarely socialize with people other than my family/I sometimes socialize with people other than my family”) and; desire to have sex with others (yes). These simple logistic regressions were separately run for positive sexual experience (i.e., answering yes to the following question: “I have had some form of sexual experiences, e.g., touching another sexually, foreplay, any sexual activity, oral sex, sexual intercourse, etc.”) and negative sexual experience (i.e., answering yes to any of the following questions: “I have agreed to have sex with someone and regretted it afterward”; “I have agreed to have sex with someone that I didn't really want to”; “I have been the victim of unwanted sexual advances or behaviors from others”; “I have initiated a sexual behavior with someone, even though I didn't really want to”). Finally, multivariate logistic regressions (enter method) were performed with significant independent variables for both positive and negative sexual experience (*p* < 0.05).

### Ethical Considerations

This study was approved by the ethical committee of the Integrated University Health and Social Services Centre of Mauricie and Centre-du-Québec (to which the Intellectual Disability and Autism Spectrum Disorder Research Institute is affiliated).

## Results

### Socializing

Significantly more participants with ASD (46.6%) than TD participants (8.7%) reported that they rarely or only sometimes socialize with people outside their family (Table 1). This finding was observed in both boys (43.9 vs. 10.3%, respectively, Table 2) and girls (48.1 vs. 8%, respectively, Table 3).

**Table 2:** Prevalence and inter-group comparisons of male adolescents/young adults with (*n* = 41) and without (*n* = 29) a diagnosis of ASD^1^.

**Items**	**With ASD agree**	**Without ASD agree**	***p***	***V***
	***n* (%)**	***n* (%)**		
**Socializing**				
I rarely or sometimes socialize with people other than my family	18 (43.9%)	3 (10.3%)	0.003	0.36^**^
**Views about romantic relationships**				
I believe that being in a long-term relationship in the future is important	31 (75.6%)	27 (93.1%)	0.06	0.23
The things that make up a good romantic relationship include if my boyfriend/girlfriend and I:				
Get along	38 (92.7%)	28 (96.6%)	0.492	0.08
Regularly speak with one another	24 (58.5%)	22 (75.9%)	0.135	0.18
Spend a lot of time together	24 (58.5%)	13 (44.8%)	0.258	0.14
Feel emotionally close with each other	23 (56.1%)	25 (86.2%)	0.008	0.32^**^
Kiss, hug, hold hands etc.	19 (46.3%)	18 (62.1%)	0.194	0.16
Engage in sexual behaviors together	13 (31.7%)	16 (55.2%)	0.05	0.24
Trust each other	32 (78%)	28 (96.6%)	0.03	0.26^*^
Are affectionate toward each other	32 (78%)	27 (93.1%)	0.09	0.20
Speak about our feelings and emotions to each other	27 (65.9%)	25 (86.2%)	0.06	0.23
Go on dates together	22 (53.7%)	20 (69%)	0.2	0.15
Have exactly the same interests	25 (61%)	7 (24.1%)	0.002	0.36^**^
Do nice things for each other so that it's fair	28 (68.3%)	19 (65.5%)	0.81	0.03
Looks after me and provides for me	28 (68.3%)	23 (79.3%)	0.31	0.12
I look after and provide for my boyfriend/girlfriend	29 (70.7%)	23 (79.3%)	0.42	0.10
Have open and honest conversations with each other	36 (87.8%)	25 (86.2%)	0.84	0.02
**Sexual interests and desires**				
I would like to have a boyfriend or girlfriend in the near future	29 (70.7%)	25 (86.2%)	0.13	0.18
I am, or have been sexually attracted toward someone	25 (61%)	29 (100%)	0.001	0.46^**^
I would like to have sex with others	25 (61%)	28 (96.6%)	0.001	0.41^**^
I want to be in a sexual relationship with another person	27 (65.9%)	28 (96.6%)	0.002	0.37^**^
I think that I will be in a sexual relationship in the future	34 (82.9%)	28 (100.0%)	0.02	0.28^*^
My level of interest in sex and sexual topics is about the same or more than other people my age	25 (61%)	28 (96.6%)	0.001	0.41^**^
I never or rarely think about sex and sexual behaviors:	13 (31.2%)	2 (6.9%)	0.013	0.30^**^
**Internet use**				
During the last month:				
I visited a web site devoted to sexual education at least once a week	2 (4.9%)	0 (0.0%)	0.228	0.14
I chatted with someone on a dating website at least once a week	2 (4.9%)	1 (3.4%)	0.771	0.04
I watch pornographic (explicit sexuality) photos or videos at least once a week	17 (41.5%)	22 (75.9%)	0.010	0.31^**^
I masturbated watching pornographic (explicit sexuality) photos or videos at least once a week	16 (39.0%)	22 (75.9%)	0.002	0.36^**^
**Sexual strategies and motivations**				
If I wanted to have casual sex (having sex with someone without being a couple or engaged in a long-term romantic relation), I would:				
Kiss them	15 (36.6%)	8 (27.6%)	0.43	0.09
Talk to them	29 (70.7%)	21 (72.4%)	0.88	0.02
Try to have the same interests as them	15 (36.6%)	5 (17.2%)	0.08	0.21
Give them something nice	16 (39%)	7 (24.1%)	0.19	0.16
Sit next to them	20 (48.8%)	13 (44.8%)	0.74	0.04
Watch them almost all of the time	5 (12.2%)	9 (31%)	0.05	0.23
Tease them to get their attention	10 (24.4%)	11 (37.9%)	0.22	0.15
Try to be around them almost all of the time	9 (22%)	7 (24.1%)	0.83	0.03
Ask them on a date	21 (51.2%)	15 (51.7%)	0.97	0.005
Tell others that I like the person	8 (19.5%)	2 (6.9%)	0.14	0.18
Tell them things about me that I think the other person will like	17 (41.5%)	6 (20.7%)	0.07	0.22
Touch them (arms, back, etc.) to show them I am interested in them	11 (26.8%)	9 (31%)	0.70	0.05
The reasons that I might have sex in the future (or have in the past) are:				
Because it feels good (Enjoyment)	21 (51.2%)	22 (75.9%)	0.037	0.25^*^
For fun	24 (58.5%)	23 (79.3%)	0.07	0.22
Because we are friends	2 (4.9%)	2 (6.9%)	0.72	0.04
Because we are in relationships	27 (65.9%)	23 (79.3%)	0.22	0.15
To show that I love them	14 (34.1%)	10 (34.5%)	0.98	0.003
Because I feel like I have to	2 (4.9%)	2 (6.9%)	0.72	0.04
Because we are married	7 (17.1%)	4 (13.8%)	0.71	0.04
Reproduction (to have children)	20 (48.8%)	18 (62.1%)	0.27	0.13
**Sexual/dyadic experience**				
I have held another person's hand (someone that you liked or you were interested in)	22 (53.7%)	25 (86.2%)	0.004	0.34^**^
I have hugged another person	33 (80.5%)	28 (96.6%)	0.048	0.24
I have kissed another person on the lips (mouth closed)	17 (41.5%)	20 (69%)	0.023	0.27^*^
I have French kissed someone (includes use of tongue)	9 (22%)	19 (65.5%)	0.001	0.44^**^
I have had some form of sexual experiences (e.g., touching another sexually, foreplay, any sexual activity, oral sex, sexual intercourse, etc.)	11 (26.8%)	21 (72.4%)	0.001	0.45^**^
Have you ever had a boyfriend or girlfriend?	17 (42.5%)	20 (69%)	0.03	0.26^*^
I enjoy using pornographic materials	20 (48.8%)	22 (75.9%)	0.023	0.27^*^
**Negative sexual experiences/worries**				
I have agreed to have sex with someone and regretted it afterward	5 (12.2%)	6 (14.6%)	0.768	0.035
I have agreed to have sex with someone that I didn't really want to	5 (12.2%)	3 (10.3%)	0.811	0.03
I have been the victim of unwanted sexual advances or behaviors from others	5 (12.2%)	4 (13.8%)	0.844	0.02
I have initiated a sexual behavior with someone, even though I didn't really want to	5 (12.2%)	2 (6.9%)	0.467	0.09
I have been teased or victimized by other people because I didn't know as much about sex as they did	6 (14.6%)	3 (10.3%)	0.597	0.06
I am worried that my sexual behaviors might be misunderstood by other people	18 (43.9%)	6 (21.4%)	0.05	0.23
I worry that I may be taken advantage of	13 (31.7%)	3 (10.7%)	0.04	0.24
**Sexual education**				
I don't know much/I would like to know better about sexuality	18 (43.9%)	2 (6.9%)	0.02	0.40^**^
I know about the same/I know much more or much about sexuality than most people my age	23 (56.1%)	27 (93.1%)	0.001	0.35^**^
It was easy for me to understand the sex-education that I received	24 (58.5%)	24 (82.8%)	0.032	0.26^*^
I would like to know more about sexuality and sexual health	25 (61%)	22 (75.9%)	0.19	0.16
Who taught you what things and actions should be done in private?				
My parents	28 (68.3%)	11 (37.9%)	0.01	0.30^**^
My brother/s or sister/s	1 (2.4%)	2 (6.9%)	0.36	0.11
My friends	11 (26.8%)	5 (17.2%)	0.35	0.11
My grandparents	4 (9.8%)	1 (3.4%)	0.31	0.12
My teachers	13 (31.7%)	7 (24.1%)	0.49	0.08
My counselor/support worker	13 (31.7%)	2 (2.9%)	0.01	0.30^**^
People on TV or movies	12 (29.3%)	7 (24.1%)	0.63	0.06
I learnt on my own/worked it out for myself	17 (41.5%)	24 (82.8%)	0.001	0.41^**^
I have mostly learnt about sex-related topics from:				
My parents	8 (20%)	4 (13.8%)	0.50	0.08
My brother/s or sister/s	1 (2.4%)	0 (0%)	0.39	0.10
My friends	1 (2.5%)	5 (17.2%)	0.03	0.26^*^
School	18 (45%)	8 (27.6%)	0.14	0.18
Reading (books, magazines)	3 (7.5%)	2 (6.9%)	0.92	0.01
Services such as counseling or community program	13 (31.7%)	2 (2.9%)	0.24	0.14
TV/movies/Internet	5 (12.5%)	4 (13.8%)	0.88	0.02
None	3 (7.5%)	4 (13.8%)	0.393	0.10
I have been taught the importance of				
Use contraception (e.g., condoms, contraceptive pill, etc.)	40 (97.6%)	29 (100%)	0.40	0.10
Having tests for sexually-transmitted infections (STI's)	37 (90.2%)	27 (93.1%)	0.67	0.05
Not making important decisions about sexual activities while affected by alcohol or drugs	34 (82.9%)	25 (86.2%)	0.71	0.04
The sexual activities that should be engaged in only in privacy are:				
Kissing someone	5 (12.2%)	0 (0%)	0.05	0.23
Touching someone in a sexual way	38 (92.7%)	24 (82.8%)	0.20	0.15
Undressing the person	28 (96.6%)	28 (97.6%)	0.80	0.03
Sexual behaviors (other than sexual intercourse)	37 (90.2%)	25 (86.2%)	0.60	0.06
Sexual intercourse	39 (95.1%)	26 (89.7%)	0.38	0.11
**Sexual identity and orientation**				
I clearly identify myself as the gender that I was born (boy or girl)	31 (75.6%)	29 (100%)	0.004^a^	0.34^**^
I consider myself as transgender^b^	2 (4.9%)	0%	0.23	0.14
I consider my sexual orientation to be:				
Heterosexual (attracted to people of the opposite sex)	26 (63.4%)	22 (75.9%)	0.269	0.13
Homosexual (attracted to people of the same sex)	5 (12.2%)	1 (3.4%)	0.198	0.15
Bisexual (attracted to people of both sex)	3 (7.3%)	3 (10.3%)	0.656	0.05
Asexual (attracted to neither sex)	3 (7.3%)	0 (0.0%)	0.137	0.18
Questioning (not sure who I am attracted to)	4 (9.8%)	3 (10.3%)	0.936	0.01

1 *Autistic Spectrum Disorder*.

*N/A, not applicable*.

a *The lowest possible p-value was set at 0.001*.

b *Reported to be transgender or did not feel that assigned gender was correct*.

* *Near medium effect size (0.25–0.29)*.

** *Medium effect size (0.30 and over)*.

**Table 3:** Prevalence and inter-group comparisons of female adolescents/young adults with (*n* = 27) and without (*n* = 75) a diagnosis of ASD^1^.

**Items**	**With ASD agree**	**Without ASD agree**	***p***	***V***
	***n* (%)**	***n* (%)**		
**Socializing**				
I rarely or sometimes socialize with people other than my family	13 (48.1%)	6 (8.0%)	0.001	0.46^**^
**Views about romantic relationships**				
I believe that being in a long-term relationship in the future is important	24 (88.9%)	68 (90.7%)	0.79	0.03
The things that make up a good romantic relationship include if my boyfriend/girlfriend and I:				
Get along	25 (92.5%)	69 (92%)	0.92	0.01
Regularly speak with one another	22 (81.5%)	64 (85.3%)	0.64	0.05
Spend a lot of time together	18 (66.7%)	31 (41.3%)	0.02	0.22
Feel emotionally close with each other	23 (85.2%)	55 (73.3%)	0.21	0.12
Kiss, hug, hold hands etc.	17 (63%)	52 (69.3%)	0.54	0.06
Engage in sexual behaviors together	11 (40.7%)	41 (54.7%)	0.22	0.12
Trust each other	25 (92.6%)	71 (94.7%)	0.69	0.04
Are affectionate toward each other	23 (85.2%)	65 (86.7%)	0.85	0.02
Speak about our feelings and emotions to each other	19 (70.4%)	66 (88.0%)	0.035	0.21
Go on dates together	18 (66.7%)	47 (62.7%)	0.71	0.04
Have exactly the same interests	19 (70.4%)	32 (42.7%)	0.014	0.24
Do nice things for each other so that it's fair	22 (81.5%)	59 (78.7%)	0.76	0.03
Looks after me and provides for me	22 (81.5%)	64 (85.3%)	0.64	0.05
I look after and provide for my boyfriend/girlfriend	21 (77.8%)	63 (84.0%)	0.47	0.07
Have open and honest conversations with each other	24 (88.9%)	73 (97.3%)	0.08	0.17
**Sexual interests and desires**				
I would like to have a boyfriend or girlfriend in the near future	24 (88.9%)	67 (89.3%)	0.95	0.01
I am, or have been sexually attracted toward someone	21 (77.8%)	71 (94.7%)	0.01	0.25^*^
I would like to have sex with others	21 (77.8%)	70 (93.3%)	0.03	0.22
I want to be in a sexual relationship with another person	14 (51.9%)	61 (81.3%)	0.003	0.30^**^
I think that I will be in a sexual relationship in the future	25 (96.2%)	70 (95.9%)	0.95	0.01
My level of interest in sex and sexual topics is about the same or more than other people my age	16 (59.3%)	67 (89.3%)	0.001	0.46^**^
I never or rarely think about sex and sexual behaviors:	7 (25.6%)	8 (10.6%)	0.013	0.30^**^
**Internet use**				
During the last month:				
I visited a web site devoted to sexual education at least once a week	2 (7.4%)	0 (0.0%)	0.017	0.24
I chatted with someone on a dating website at least once a week	3 (11.1%)	3 (4%)	0.19	0.13
I watch pornographic (explicit sexuality) photos or videos at least once a week	4 (14.8%)	12 (16%)	0.89	0.01
I masturbated watching pornographic (explicit sexuality) photos or videos at least once a week	4 (14.8%)	10 (13.3%)	0.85	0.02
**Sexual strategies and motivations**				
If I wanted to have casual sex with someone, I would (casual sex is having sex with someone without being a couple or engaged in a long-term romantic relation):				
Kiss them	6 (22.2%)	28 (37.3%)	0.15	0.14
Talk to them	21 (77.8%)	45 (60.0%)	0.10	0.16
Try to have the same interests as them	7 (25.9%)	5 (6.7%)	0.008	0.26^*^
Give them something nice	6 (22.2%)	3 (4.0%)	0.004	0.28^*^
Sit next to them	10 (37.0%)	23 (30.7%)	0.54	0.06
Watch them almost all of the time	5 (18.5%)	15 (20.0%)	0.87	0.02
Tease them to get their attention	5 (18.5%)	37 (49.3%)	0.005	0.28^*^
Try to be around them almost all of the time	6 (22.2%)	15 (20.0%)	0.81	0.02
Ask them on a date	8 (29.6%)	39 (52.0%)	0.046	0.20
Tell others that I like the person	5 (18.5%)	18 (24.0%)	0.56	0.06
Tell them things about me that I think the other person will like	10 (37.0%)	12 (16.0%)	0.02	0.23
Touch them (arms, back, etc.) to show them I am interested in them	8 (29.6%)	27 (36.0%)	0.55	0.06
The reasons that I might have sex in the future (or have in the past) are:				
Because it feels good (Enjoyment)	12 (44.4%)	61 (81.3%)	0.001	0.36^**^
For fun	12 (44.4%)	58 (77.3%)	0.002	0.31^**^
Because we are friends	0 (0%)	6 (8%)	0.13	0.15
Because we are in relationships	18 (66.7%)	63 (84%)	0.056	0.19
To show that I love them	8 (29.6%)	23 (30.7%)	0.92	0.01
Because I feel like I have to	2 (7.4%)	3 (4.0%)	0.48	0.07
Because we are married	1 (3.7%)	15 (20%)	0.046	0.20
Reproduction (to have children)	6 (22.2%)	39 (52.0%)	0.008	0.27^*^
**Sexual/dyadic experience**				
I have held another person's hand (someone that you liked or you were interested in)	25 (92.6%)	68 (90.7%)	0.76	0.03
I have hugged another person	27 (100%)	73 (97.3%)	0.39	0.09
I have kissed another person on the lips (mouth closed)	21 (77.8%)	67 (89.3%)	0.14	0.15
I have French kissed someone (includes use of tongue)	16 (63%)	62 (82.7%)	0.04	0.21
I have had some form of sexual experiences (e.g., touching another sexually, foreplay, any sexual activity, oral sex, sexual intercourse, etc.)	16 (59.3%)	64 (85.3%)	0.005	0.28^*^
Have you ever had a boyfriend or girlfriend?	22 (81.5%)	62 (82.7%)	0.89	0.01
I enjoy using pornographic materials	12 (44.4%)	36 (48%)	0.75	0.03
**Negative sexual experiences/worries**				
I have agreed to have sex with someone and regretted it afterward	12 (44.4%)	30 (40%)	0.69	0.04
I have agreed to have sex with someone that I didn't really want to	13 (48.1%)	31 (41.3%)	0.54	0.06
I have been the victim of unwanted sexual advances or behaviors from others	14 (51.9%)	24 (32%)	0.07	0.18
I have initiated a sexual behavior with someone, even though I didn't really want to	11 (40.7%)	19 (25.3%)	0.13	0.15
I have been teased or victimized by other people because I didn't know as much about sex as they did	7 (25.9%)	11 (14.7%)	0.19	0.13
I am worried that my sexual behaviors might be misunderstood by other people	8 (30.8%)	11 (15.1%)	0.08	0.18
I worry that I may be taken advantage of	13 (50.0%)	31 (42.5%)	0.51	0.07
**Sexual education**				
I don't know much/I would like to know better about sexuality	11 (40.7%)	14 (18.7%)	0.02	0.23
I know about the same/I know much more or much about sexuality than most people my age	27 (59.3%)	61 (81.3%)	0.04	0.22
It was easy for me to understand the sex-education that I received	9 (33.3%)	59 (78.7%)	0.000	0.42^**^
I would like to know more about sexuality and sexual health	14 (51.9%)	41 (54.7%)	0.80	0.03
Who taught you what things and actions should be done in private?				
My parents	14 (51.9%)	42 (56.0%)	0.71	0.04
My brother/s or sister/s	3 (11.1%)	3 (4.0%)	0.18	0.13
My friends	5 (18.5%)	25 (33.3%)	0.15	0.14
My grandparents	0 (0.0%)	4 (5.3%)	0.22	0.12
My teachers	11 (40.7%)	18 (24.0%)	0.10	0.16
My counselor/support worker	6 (22.2%)	6 (8.0%)	0.049	0.19
People on TV or movies	10 (37.0%)	24 (32.0%)	0.63	0.05
I learnt on my own/worked it out for myself	15 (55.6%)	44 (58.7%)	0.78	0.03
I have mostly learnt about sex-related topics from:				
My parents	4 (14.8%)	8 (10.7%)	0.57	0.06
My brother/s or sister/s	1 (3.7%)	0 (0%)	0.09	0.17
My friends	4 (14.8%)	26 (34.7%)	0.05	0.19
School	8 (29.6%)	17 (22.7%)	0.47	0.07
Reading (books, magazines)	3 (11.1%)	4 (5.3%)	0.31	0.10
Services such as counseling or community program	1 (3.7%)	1 (1.3%)	0.47	0.08
TV/movies/Internet	3 (11.1%)	11 (14.7%)	0.65	0.05
None	1 (3.7%)	4 (5.3%)	0.74	0.03
I have been taught the importance of:				
Use contraception (e.g., condoms, contraceptive pill, etc.)	26 (96.3%)	75 (100.0%)	0.09	0.17
Having tests for sexually-transmitted infections (STI's)	23 (85.2%)	69 (92.0%)	0.31	0.10
Not making important decisions about sexual activities while affected by alcohol or drugs	23 (85.2%)	66 (88.0%)	0.71	0.04
The sexual activities that should be engaged in only in privacy are:				
Kissing someone	3 (11.1%)	2 (2.7%)	0.08	0.17
Touching someone in a sexual way	25 (92.6%)	64 (85.3%)	0.33	0.10
Undressing the person	26 (96.3%)	71 (94.7%)	0.77	0.03
Sexual behaviors (other than sexual intercourse)	24 (88.9%)	67 (89.3%)	0.95	0.006
Sexual intercourse	25 (92.6%)	67 (89.3%)	0.63	0.05
**Sexual identity and orientation**				
I clearly identify myself as the gender that I was born	19 (70.4%)	70 (93.3%)	0.002^a^	0.30^**^
I consider myself as transgender^b^	4 (14.8%)	2 (2.7%)	0.02	0.23
I consider my sexual orientation to be:				
Heterosexual (attracted to people of the opposite sex)	17 (63.0%)	52 (69.3%)	0.54	0.06
Homosexual (attracted to people of the same sex)	1 (3.7%)	0 (0.0%)	0.09	0.17
Bisexual (attracted to people of both sex)	5 (27.9%)	17 (22.7%)	0.73	0.03
Asexual (attracted to neither sex)	1 (3.7%)	1 (1.3%)	0.45	0.08
Questioning (not sure who I am attracted to)	1 (3.7%)	5 (6.7%)	0.58	0.06

1 *Autistic Spectrum Disorder*.

*N/A, not applicable*.

a *The lowest possible p-value was set at 0.001*.

b *Reported to be transgender or did not feel that assigned gender was correct*.

* *5Near medium effect size (0.25–0.29)*.

** *Medium effect size (0.30 and over)*.

### Views About Romantic Relationships

Much more similarities than differences were observed between the groups (ASD vs. TD) concerning their views about romantic relationships (Table 1). The notable exception was the significantly greater importance given by participants with ASD to have exactly the same interests with the other person to make up a good romantic relationship compared with their TD peers (64.7 vs. 37.5%, respectively, Table 1).

### Sexual Interests and Desires

Both groups wish to have a boyfriend/girlfriend in similar (not significantly different) proportions (ASD: 77.9% and TD: 82.7%; Table 1). The majority of participants with ASD have been sexually attracted toward someone at least once (67.6%), they wish to have sex with another person (67.6%), and they want to be in a sexual relationship (60.3%; Table 1). However, these rates were all significantly lower than those found in TD participants (96.2, 94.2, and 85.6%, respectively; Table 1). These differences in rates of desire applied to boys (Table 2) and girls (Table 3), although the difference for wishing to have sex with another person did not reach statistical significance between girls. Similarly, a significantly lower proportion of participants with ASD felt that their interest in sex was equal or greater to their peers (60.3%) compared with TD participants (91.3%; Table 1). Accordingly, a significantly higher proportion of participants with ASD (29.4%) than TD participants (9.6%) reported they never or rarely think about sex (Table 1). These results applied to both boys (Table 2) and girls (Table 3).

### Pornography Consumption

As for Internet pornography consumption, a significantly lower proportion of boys with ASD reported watching pornography (41.5 vs. 75.9%, respectively) and/or masturbating with pornography (39 vs. 75.9%, respectively) regularly (at least once a week) compared with those without ASD (Table 2). Among girls, rates of pornography consumption were much lower in both groups, without difference between them (Table 3).

### Sexual Strategies and Motivations

Significant differences between participants with ASD vs. TD participants were found concerning strategies they would use to have casual sex. Whereas significantly higher proportions of the former group would act as a function of the other person (i.e., try to have similar interest as them and give them something nice), more TD participants stated that they would tease them to get attention (Table 1). These results held true for girls (Table 3) and partially for boys (only for trying to have same interests; Table 2). A significantly higher proportion of girls with ASD (39.7%) compared to TD girls (17.3%) also stated that they would tell things about themselves that they think the other person would like in order to have casual sex (Table 3).

Similarly, significantly fewer participants with than without ASD reported that they would have sex because it feels good (48.5 vs. 79.8%, respectively) or for fun (52.9 vs. 77.9%, respectively; Table 1). These results were confirmed for both boys (Table 2) and girls (Table 3).

### Sexual/Dyadic Experiences

Significantly lower proportions of participants with ASD had experience with a variety of sexual/dyadic behaviors (Table 1). However, these differences mostly concerned boys (Table 2), as no difference was found between girls with and without ASD except for sexual experience (oral sex, sexual intercourse, etc.; Table 3). For instance, although a significantly lower percentage of boys with (42.5%) than without (69%) ASD ever had a girlfriend/boyfriend (Table 2), no difference emerged between the groups of girls (Table 3). In fact, a higher percentage of girls with ASD (81.5%) than boys without ASD (69%) reported having had a boyfriend/girlfriend at least once in their lifetime.

### Negative Sexual Experience/Worries

No significant difference emerged between the groups (participants with vs. without ASD) for rates of negative sexual experiences (Table 1). However, much higher proportions of girls overall reported negative sexual experiences (between 25.3 and 51.9%, Table 3) compared with boys (between 7 and 14.6%, Table 2). The proportion of girls reporting unwanted sexual advances or behaviors from others was higher among those with ASD (51.9%) than among TD girls (32%), although the difference did not reach statistical significance. These results might reflect the high base rate of sexual victimization among girls in general, a lack of statistical power or both. More participants with ASD also worried that their sexual behaviors might be misunderstood by other people compared with their TD peers (38.8 vs. 16.8%, respectively, Table 1).

### Sexual Education and Knowledge

Significantly higher rates of participants with ASD felt they did not know much about sexuality (42.6%) than TD participants (15.4%; Table 1). Therefore, significantly more TD participants considered they know as much or more about sexuality than their peers (84.6%) compared with participants with ASD (57.4%; Table 1). In addition, a significantly lower proportion of participants with ASD (48.5%) found it easy to understand sex education compared with TD participants (79.9%; Table 1). However, the majority of both groups expressed wishes to know more about sexuality (no significant difference between the groups; Table 1). These results held for boys (Table 2) and partially for girls (Table 3). As expected, sexual education and advice was more likely to be given by counselors and less likely by friends to participants with ASD compared with their TD peers (Table 1). These results only concerned boys, however, as no difference emerged between groups of girls (Table 3). In fact, boys with ASD were more likely to have received sexual education or advice by their parents or counselors, and less likely to receive it from friends or by themselves than boys without ASD (Table 2).

Information about contraception, sexually-transmitted infections, and usage of alcohol/drugs seem to have been adequately provided in both groups (Table 1). Similarly, no significant difference emerged between participants with ASD and TD participants concerning their knowledge of behaviors requiring privacy (Tables 1–3).

### Sexual Identity and Orientation

Significantly lower rates of participants with ASD (73.5%) reported that they identify as their assigned gender compared with TD participants (95.2%, Table 1). This finding was true for both males (73.5 vs. 100%, respectively, Table 2) and females (70.4 vs. 93.3%, respectively, Table 3). However, no significant differences emerged between the groups for rates of any sexual orientation (Table 1). This finding was also true for both males (Table 2) and females (Table 3).

### Predictions of Positive and Negative Sexuality

Gender [female; Wald = 6.8, *p* = 0.009, Exp(B) = 4.0, C.I.: 1.4–11.1], age at first diagnosis for ASD [older; Wald = 6.2, *p* = 0.01, Exp(B) = 1.1, C.I.: 1.03–1.2], self-reported level of knowledge about sexuality and sexual behaviors [higher; Wald = 4.9, *p* = 0.03, Exp(B) = 3.3, C.I.: 1.2–9.5], and desire to have sex with others [yes; Wald = 7.9, *p* = 0.005, Exp(B) = 6.9, C.I.: 1.8–26.6] were all significant single predictors of having positive sexual experience among participants with ASD. Thus, current age and extra-familial sociability were not associated with the odds of having positive sexual experience among persons with ASD. Unexpectedly, the exact same variables were also significant predictors of having negative sexual experience among participants with ASD: Being a girl [Wald = 6.8, *p* = 0.009, Exp(B) = 4.0, C.I.: 1.4–11.1], being older at first diagnosis of ASD [Wald = 8.0, *p* = 0.005, Exp(B) = 1.2, C.I.: 1.0–1.3], considered as having good knowledge about sexuality and sexual behaviors [Exp(B) = 4.5, *p* = 0.007, C.I.: 1.5–13.4], and desiring having sex with others [Wald = 5.7, *p* = 0.01, Exp(B) = 4.5, C.I.: 1.3–15.3]. Current age and extra-familial sociability were not associated with the odds of having negative sexual experience. Multivariate logistic regressions conducted with these four significant variables indicated that two of them contributed significantly and independently to the variance of having positive sexual experience: older age at first diagnosis for ASD and better knowledge about sexuality and sexual behaviors. Together, these two variables explained approximately a fifth of the variance (22%) and correctly classified 72% of the sample (*R*^2^ = 0.223). Again, the same two variables significantly and independently predicted the presence of negative sexual experience, explaining approximately a third of the variance and correctly classified 73.4% of the sample (*R*^2^ = 0.353). To obtain the most parsimonious regression models possible, independent variables that did not significantly contribute to the explanation of the dependent variables were excluded. Although more contingent upon a specific sample, this procedure allows for the reduction of the number of variables in the model and results in a more statistically stable model (72). After gender and desire to have dyadic sex were excluded from analyses, final logistic regressions were conducted for both positive and negative sexual experiences. The interaction effects were determined for each of the models but were not retained in the final analysis, as none were significant.

These unexpected findings that positive and negative sexual experience were predicted by the same variables might reflect at least two possibilities: (1) Participants with ASD who have positive sexual experience also tend to have negative sexual experience; (2) Participants with ASD interpreted questions meant to assess positive sexual experience (i.e., the dependent variable: “I have had some form of sexual experiences, e.g., touching another sexually, kissing, foreplay, any sexual activity, oral sex, sexual intercourse, etc.”) as questions related to any sexual experience (i.e., unwanted oral sex). In order to assess these two alternatives, rates of positive vs. negative sexual experience were compared between and within groups. Among TD participants, 81.7% (*n* = 85) had at least one positive sexual experience and 53.8% (*n* = 56) had at least one negative sexual experience, with 36.5% (*n* = 31) reporting only positive experience. Among participants with ASD, 39.7% (*n* = 27) reported at least one positive sexual experience but 39.7% (*n* = 27) also reported at least one negative sexual experience, with only 18.5% (*n* = 5) reporting only positive sexual experience (another 5 participants reported only negative sexual experience). Therefore, adolescents/young adults with any sexual experience tended to report some negative sexual experience, especially among those with ASD.

## Discussion

The main goal of this exploratory study was to ask adolescents/young adults with ASD about their sexual knowledge, desire and experience. A second study goal was to compare their responses to those of TD peers. The third goal was to compare results between boys and girls in both groups. A final objective was to assess the predictive value of selected variables on positive and negative sexual experience among participants with ASD.

As expected, the majority of our participants expressed the desire to have romantic/sexual relationships. Still, it is also worth noting that significant proportions of adolescents/young adults with ASD reported low or no interest in sexual relationships, among both boys and girls. These results illustrate the heterogeneity of the group and the fact that some persons with ASD, at least when young adults, are simply not interested in socio-sexual behaviors. Even weekly rates of watching pornography and masturbating were lower in boys with ASD in this study. Compared with TD participants, adolescents/young adults with ASD also reported less sexual knowledge and experience. For instance, higher proportions of boys and girls with ASD felt that trying to have common interests with another person or giving that person something nice were good strategies to have casual sex with them. Furthermore, fewer participants with ASD had experience with actual sexual behaviors (e.g., oral sex, intercourse). These results stress the importance of providing adequate and adapted sexual education to adolescents/young adults with (and without) ASD. First, adolescents with ASD receive less sexual information from their friends compared with TD adolescents. Although biological aspects of sexuality seem to be well-understood (e.g., contraception, STI), socio-sexual education is needed. In fact, most participants in this study, with or without ASD, wished to learn more about sexuality. Second, however, more sexual knowledge is also associated with higher risk to be sexually victimized in young persons with ASD. Therefore, socio-sexual education should go beyond physical aspects of sexuality and encompass such notions as intimacy, self-respect, self-esteem, mutual consent, understanding intentions of others, and verbal and non-verbal romantic communication.

Knowledge about romantic relationships (e.g., things that make up a good relationship) and privacy was generally considered as good by participants in this study, contrary to what is typically reported by parents or among adults with ASD (13, 15). Similar conclusions were drawn from two other self-report studies of adolescents with ASD (18, 23). These results might reflect the influence of using self-reports (instead of parent or caregiver reports), better social services offered to youngsters with ASD nowadays, a better access to information through Internet, or a mix of these factors.

Interestingly, substantial differences emerged between boys and girls with ASD in this study, reflecting the importance of considering both genders separately in the sexual investigation of persons with ASD. As in previous studies of adults (10, 38), girls with ASD reported better knowledge and experience with romantic/sexual relationships than boys. Despite better knowledge, girls were also much more likely to report past negative sexual experiences than boys, although these results applied to all girls (with or without ASD). The rate of girls with ASD reporting negative sexual experience was alarmingly high in this study (51.9%), yet somewhat lower than that found by Pecora et al. [(30); from 60 to 78.2% for women with ASD depending on specific items].

Also noteworthy is the fact that significantly lower rates of participants with ASD, both among boys and among girls, clearly identified themselves with their birth gender compared with TD participants, extending data obtained with adults (26, 27). This gender diversity might reflect psychological (e.g., greater openness) and/or neurological factors. For instance, prenatal androgens are believed to masculinize the brain and a negative association exists between intrauterine exposition to testosterone (high) and the 2:4 digit (major and auricular fingers) ratio [low; (73)]. Given that persons with ASD have, on average, lower 2:4 digit ratios (74, 75), and that higher steroidogenic activity is found in amniotic fluid during pregnancy leading to boys with ASD (76, 77), high levels of androgens *in utero* are hypothesized to contribute to ASD (76). Although that hypothesis remains controversial [e.g., (78)], it might be associated with the intriguing link between ASD and gender dysphoria because rates of gender dysphoria or transgender are both significantly higher among persons with ASD and linked to lower 2:4 digit ratios (79, 80). Future neurobiological studies of ASD should further explore these avenues.

As for sexual orientation, rates of homosexuality or bisexuality did not differ between ASD vs. non-ASD groups in the present study, contrary to what is usually reported in adults with ASD [(38, 47, 49–51, 54); see (26, 27) for reviews]. It should be noted, however, that base rates of non-heterosexuality were significantly higher in the TD group, both for boys and girls (between 25 and 30%) than among the general population of adults in Canada [between 1 and 3%; (81)]. This result might reflect the relatively low age of participants in this study and the high proportions still unsure of (or more open about) their sexual orientation in both groups.

As expected, being female, being older at first diagnosis, having a better knowledge about sexuality and sexual behaviors, and desiring to have sex with others were all significant predictors of having positive sexual experience among participants with ASD. What was less expected, however, was the fact that the same variables were also significant predictors of negative sexual experience, especially for having a better sexual knowledge. Being older at first diagnosis of ASD and having better sexual knowledge might be related with better capacities to mask (or camouflage) difficulties in social interaction and communication, particularly among girls (82, 83). These characteristics, paired with a desire to have sex with others (this study), a strong need to be liked by peers and better mimicking and vocabulary capacities [e.g., (84)], might put these girls at higher risks to be misinterpreted, exploited, and/or sexually abused (40). Future investigations should test this possible link between symptom masking and negative sexual experience.

Finally, some limitations of this study should be noted before generalizing these results. First, dividing study groups by genders significantly lowered the statistical power in this study. Further confirmatory studies with larger groups of male and female participants and higher statistical power are warranted. Second, the diagnoses of ASD were not confirmed with a screening tool [e.g., AQ, (85)] in this study. Although all participants with ASD received an official diagnosis confirmed with self-report, it remains possible that some of them would not have met the cut-off of a screening instrument. In opposition, some participants of the control group might have presented autistic traits. Third, although the questionnaire (SBS-III) has been statistically validated (70), its face validity was not assessed and some questions related to sexuality might have been difficult to understand for certain participants. For instance, although some questions about sexual experience were aimed at documenting positive sexuality (“I have had some form of sexual experiences, e.g., touching another sexually, kissing, foreplay, any sexual activity, oral sex, sexual intercourse, etc.), they might have been interpreted as comprising any sexual experience, including non-consenting behaviors. Therefore, the definition of positive sexuality in this study might in fact have included negative experience. Clearly, further validation studies are required with the SBS-III, including pilot testing involving feedback and suggestions from young persons with ASD. Finally, all items of the questionnaire were dichotomized (e.g., yes-no) for statistical analyses so that nuance and some information were lost in the process.

Overall, results from this study confirm that most adolescents/young adults with ASD wish to be in a romantic and/or sexual relationship, although a significant minority report lower or no interest for sex. Girls with ASD have more romantic and sexual experience than boys with ASD. However, the rate of negative sexual experience was particularly high among girls with ASD, associated with a later age of diagnosis and better sexual knowledge. It is possible that better capacities to mask difficulties in social interactions and communication, paired with a need to be liked and a desire to be in a romantic/sexual relationship represent risk factors for sexual victimization of young adults with ASD. Sexual education should not only be provided to adolescents with and without ASD, it should also include socio-sexual notions to prevent this type of victimization and promote a healthy sexual life.

## Data Availability Statement

The raw data supporting the conclusions of this article will be made available by the authors, without undue reservation.

## Ethics Statement

The studies involving human participants were reviewed and approved by Integrated University Health and Social Services Centre of Mauricie and Centre-du-Québec. Written informed consent to participate in this study was provided by the participants' legal guardian/next of kin.

## Author Contributions

CCJ, JC, SM, CN, and M-HP designed the study, adapted the instrument, and collected the data. M-HP supervised the recruitment of participants. CCJ, JC, and SM conducted statistical analyses. CCJ wrote the first draft of the manuscript. JC, SM, CN, and M-HP revised the manuscript. All authors contributed to the article and approved the submitted version.

## Conflict of Interest

The authors declare that the research was conducted in the absence of any commercial or financial relationships that could be construed as a potential conflict of interest.
